# Genomics is changing personal healthcare and medicine: the dawn of iPH (individualized preventive healthcare)

**DOI:** 10.1186/s40246-015-0052-0

**Published:** 2015-11-04

**Authors:** Ruty Mehrian-Shai, Juergen K. V. Reichardt

**Affiliations:** Pediatric Hemato-Oncology, Chaim Sheba Medical Center, Ramat Gan, Israel; Division of Tropical Health and Medicine, James Cook University, Townsville, QLD Australia; Present Address: Yachay Tech University, San Miguel de Urcuquí, Ecuador

**Keywords:** Human genomics, Individual information, Personalized medicine, Medical education, Health care cost

## Abstract

This opinion piece focuses on the convergence of information technology (IT) in the form of personal monitors, especially smart phones and possibly also smart watches, individual genomic information and preventive healthcare and medicine. This may benefit each one of us not only individually but also society as a whole through iPH (individualized preventive healthcare). This shift driven by genomic and other technologies may well also change the relationship between patient and physician by empowering the former but giving him/her also much more individual responsibility.

Costs for healthcare in most countries are rising rapidly and account for a sizeable fraction of a country’s GDP (gross domestic product) [1]. This trend is most evident in the USA where the fraction of GDP spent on healthcare has doubled from 8.2 % in 1980 to 16.2 % in 2012 [1]. This generally rising trend is noticeable in Australia as well [1], although it is not as pronounced with an increase from 5.8 % of GDP in 1980 to 8.6 % in 2011. Clearly, this escalation is not sustainable and hence cannot continue indefinitely. Healthcare must be sustainable. In fact, a significant burden is expended towards the end of life [2] suggesting that a more preventive approach may be beneficial.

We propose here that a convergence of information technology epitomized by individual monitors, incl. smart phones and smart watches, and genomics in the form of personal genomic information, especially on disease susceptibility, will result in new health information accessible to each individuum.

The four converging areas leading to what we propose to call individualized preventive healthcare (iPH) are:

First, ongoing rapid advances in personal monitors, e.g. monitoring heart rate or tracking day to day activity, e.g. smart phones and smart watches allow individuals to collect, monitor and collate relevant health information personally. These data can then be analyzed through online world-wide searches, e.g. “Googling”, by the patient him/herself before seeing a physician. There are also significant ethical issues associated with these new developments [3] which must be carefully considered and addressed.

Furthermore, genome sequencing is now approaching a cost of just $1000 [4]. This price, which is continuously falling, will put one’s own whole human genome DNA sequence and its information at individual fingertips. Clearly, such genomic disease-related risk information must be accompanied by appropriate and careful interpretation and counselling [5]. In any case, individual genomic information can be used to identify risks which can then be mitigated if not eliminated altogether. Of course, these developments in genomic science again put the patient at the very heart of the matter by allowing him/her to search for information, e.g. by Googling, before seeing a physician to prevent (or at least slow) disease.

Third, the microbiome [6] which is intrinsically personal and largely determined genomically also has become of considerable interest and will find its way into modern medical practice, perhaps again by patients Googling information. In fact, because of the significant role of the gut microbiota in human physiology and disease [6], new and unique opportunities will arise for personal control of the gut flora. This will result in novel strategies to prevent and treat diseases including cancer, inflammatory bowel disease (IBD), diabetes, heart disease, allergy and perhaps even mental illness. The pathogenesis of disease can be influenced also by various epigenomic factors: microbiota, food intake, stress level and physical activity. All these factors can be monitored, investigated and evaluated.

We also note that the US NIH/NCI initiative on personalized medicine [7] to accelerate precision medicine and the plan to monitor genetic and environmental factors of “cohort” of 1 million or more Americans will set the basis of the multifactorial disease “warning” machinery and provide valuable new insights.

Lastly, there is also an urgent need for credible and trusted sources of medical information on the Internet for individual patients to access and inform themselves. This important issue has been addressed already, e.g. [8], but will require constant attention, especially from all of us, the medical professionals. Similarly, relationships with patients are apt to change if they “arm” themselves with Googled information.

## Conclusion

In conclusion, we believe that iPH (individualized preventive healthcare) which arises from a convergence of personal monitors, incl. information technology (IT), genomics, incl. the microbiome and vastly expanded information available online will offer not only great individual benefits by improving health through personalized information and prevention but also significant cost savings in the long run for healthcare. Furthermore, iPH may radically alter the relationship between physicians and patients. This will give patients not only increased information but also significant individual responsibility. Future research, education and thoughtful discourse should prepare individuals, medical practitioners, scientists, (health) economists if not societies at large for these important changes.

## Abbreviations

GDP: gross domestic product
iPH: individualized preventive healthcare
IT: information technology
